# Zukunftsperspektiven der Deutschen Gesellschaft für Chirurgie 2022

**DOI:** 10.1007/s00104-022-01794-6

**Published:** 2022-12-27

**Authors:** Hauke Lang

**Affiliations:** Klinik für Allgemein‑, Viszeral- und Transplantationschirurgie, Universitätsmedizin der Johannes Gutenberg-Universität Mainz, Langenbeckstr. 1, 55131 Mainz, Deutschland

**Keywords:** Geschichte, Fachgesellschaften, Dachgesellschaft, Aufgabenprofil, Interdisziplinarität, History, Specialist society, Parent society, Task profile, Interdisciplinarity

Die Deutsche Gesellschaft für Chirurgie (DGCH) blickt auf eine 150-jährige Geschichte zurück. 150 Jahre DGCH bedeuten eine Erfolgsgeschichte, geprägt von Innovationen und chirurgischen Pionierleistungen, geprägt aber auch von Veränderungen in der Struktur der DGCH. Die Chirurgie hat sich glänzend entwickelt und ist heute eine der wesentlichen Säulen des Gesundheitssystems. Die zunehmende Spezialisierung führte dazu, dass sich die Chirurgie immer weiter aufgliederte und eigene Fachgesellschaften gegründet wurden.

Klinisch ließen sich diese Spezialisierungen noch lange Zeit innerhalb einer breit aufgestellten Fachgesellschaft abbilden. Erst in den letzten Jahrzehnten hat sich die DGCH, die bei ihrer Gründung noch die wissenschaftliche Gesellschaft aller chirurgisch tätigen Ärzte war, zunehmend weg von einer Fach- und hin zu einer Dachgesellschaft entwickelt. Dieser Wandel implizierte eine Verschiebung des Aufgabenprofils und der Zuständigkeiten der DGCH und berührte auch ihr Selbstverständnis. Während die fachlichen Verantwortlichkeiten und die Autonomie der einzelnen chirurgischen Fachgesellschaften unangetastet blieben, rückte und rückt für die DGCH die gemeinsame Interessenvertretung im übergeordneten Sinne zunehmend in den Vordergrund. Dies trifft auf alle fachübergreifenden Themen wie Forschungsförderung, Versorgungsforschung, Qualitätssicherung oder Patientensicherheit zu, aber auch auf Aufgaben wie z. B. die Vertretung übergeordneter chirurgischer Interessen gegenüber der Bundesärztekammer oder den Organen der Selbstverwaltung im Gesundheitswesen, dem Gemeinsamem Bundesausschuss oder dem Gesundheitsministerium und schließlich auch gegenüber anderen Fachgebieten. Zudem vertritt die DGCH die Chirurgie in Verbänden wie der Arbeitsgemeinschaft der Wissenschaftlichen Medizinischen Fachgesellschaften e. V. (AWMF) oder Anstalten des öffentlichen Rechts, beispielsweise dem Institut für medizinische und pharmazeutische Prüfungsfragen (IMPP). Für eine wirkungsvolle Vermittlung chirurgischer Standpunkte und Vertretung chirurgischer Interessen anderen medizinischen Fachdisziplinen gegenüber, aber auch für den Dialog mit Politik und Gesellschaft ist dabei eine gemeinsame Chirurgische Gesellschaft unabdingbar.

Die DGCH hat mit ihren über 20.000 Mitgliedern sowohl das Mandat als auch das Format, um diesen Aufgaben nachzukommen.

Die DGCH steht heute an einer Schwelle zu einem neuen Entwicklungsschritt, weniger, weil sie auf genau 150 Jahre DGCH zurückblickt und ein neues halbes Jahrhundert beginnt, sondern weil wir es zusätzlich zu den rasanten technischen und wissenschaftlichen Entwicklungen unseres Faches auch mit den Folgeerscheinungen der pandemischen Krise zu tun haben, die wir durchlebt haben und noch immer durchleben.

Die Pandemie hat im Gesundheitswesen viele Defizite offengelegt, beispielsweise im Bereich der Digitalisierung und der Verteilung von Ressourcen, fehlende Netzwerkstrukturen oder überkommene, nicht mehr zeitgemäße Strukturen sowohl in der Patientenversorgung als auch in der Forschung. Aber die Pandemie hat nicht nur Defizite schonungslos offenbart, sondern sie war vielfach auch Katalysator für neue Entwicklungen, sie hat Prozesse angestoßen oder beschleunigt.

Schon Winston Churchill wusste um die Chancen, die einer Krise erwachsen, als er sagte:

„Never let a good crisis go to waste!“

Auch die Deutsche Gesellschaft für Chirurgie darf die sich durch die Krise auftuenden Chancen nicht ungenutzt verstreichen lassen.

Aber es ist nicht nur die Pandemie, hinzu kommen auch neue politische, soziale, gesellschaftliche und ökonomische Rahmenbedingen sowie auch längst hinter uns geglaubte geopolitische Veränderungen, die Einfluss auf unsere Gesellschaft und auf unser Gesundheitssystem haben. Dies alles sowie der schon erwähnte rasante technische und wissenschaftliche Fortschritt fordern eine Veränderung des chirurgischen Alltags und der Chirurgie und damit auch eine Veränderung der DGCH in ihrer Struktur, Ausrichtung und Zielsetzung.

Es ist dies allerdings nicht der erste Umbruch, den die deutsche Gesellschaft für Chirurgie seit ihrer Gründung erlebt und gestaltet. Die Entwicklung der Chirurgie und der DGCH ist nicht immer kontinuierlich verlaufen, wiederholt kam es zu mitunter gar sprunghaften Veränderungen.

Wie gravierend diese anstehenden Veränderungen für die DGCH sein werden, wird ganz entscheidend davon abhängen, wie sehr sich Chirurginnen und Chirurgen an diesem Wandel beteiligen. Oder, wie es Karl Heinrich Bauer, einer der Pioniere der onkologischen Chirurgie in Deutschland, in seiner Präsidentenrede 1952 in München formuliert hat:

„Was wir nicht selber tun, das wird mit uns getan.“

Die momentan wichtigsten Handlungsfelder, neben der Digitalisierung und den fachlichen Herausforderungen, betreffen

- die fortschreitende Interdisziplinarität mit dem Verwischen medizinischer Fächergrenzen,
- die Situation der chirurgischen Forschung,
- die Nachwuchsgewinnung und Nachwuchsförderung
- sowie die unvermindert zunehmende Ökonomisierung der Chirurgie wie auch der gesamten Medizin.

## Interdisziplinarität

Zur Wahrung der Ganzheitlichkeit der Behandlung unserer Patienten erfordert die zunehmende Spezialisierung eine verstärkte Interdisziplinarität als Gegengewicht. Längst sind Chirurginnen und Chirurgen Akteurinnen und Akteure in einem multidisziplinären Team geworden, eingebunden und verflochten in Organzentren oder in übergeordneten interdisziplinären Einheiten. Das Zusammenspiel mit anderen Disziplinen eröffnet neue Möglichkeiten und Indikationsfelder. Chirurgie wird in Zukunft an manchen Stellen weniger, an anderen mehr und an vielen sicher anders gebraucht werden. Das Zusammenspiel der Disziplinen bietet Chancen, aber es birgt auch die Gefahr eines Verlustes der Sichtbarkeit der chirurgischen Fachlichkeit, vielleicht sogar eines Verlustes an chirurgischer Identität.

Dem muss die DGCH entgegenwirken – nicht so sehr, weil der Verlust an Sichtbarkeit am chirurgischen Selbstverständnis nagt, sondern weil er einen Verlust an Attraktivität bedeutet und damit zu einem Nachteil beim Werben um Nachwuchskräfte und bei der Vergabe von Forschungs- und Fördergeldern führt.

## Forschung

Forschung nimmt in der Medizin einen immer größeren Stellenwert ein, und Forschung ist auch für die Zukunft der Chirurgie essenziell.

„Wer aufhört besser zu werden, hat aufgehört, gut zu sein.“

Dieses Zitat von Oliver Cromwell ist ebenso zeitlos wie uneingeschränkt gültig. Ein Verharren auf dem Status quo bedeutet Rückschritt und einen Verlust an Bedeutung. Nur wenn die Chirurgie noch wissenschaftlicher wird, kann sie auf Augenhöhe mit anderen Fachdisziplinen bestehen.

Wenngleich chirurgische Forschung in erster Linie klinisch und auch angewandt-technisch ist, so muss sich die Chirurgie auch in der Grundlagen- und translationalen Forschung aktiver beteiligen. „From bench to bedside“ – dabei ist nicht der Weg das Ziel, sondern der erfolgreiche klinische Einsatz eines Produktes am Ende eines langen Forschungs- und Entwicklungsprozesses. Die Chirurgie ist hier viel mehr als nur Materiallieferant und Erfüllungsgehilfe, sie muss sich als integralen Partner in der translationalen Forschungskette verstehen und soll auch so wahrgenommen werden.

Mit der „Sektion Chirurgische Forschung“ und der „Sektion minimal-invasive, Computer- und Telematik-assistierte Chirurgie“ besitzt die DGCH zwei sich über alle Fachdisziplinen erstreckende Forschungsplattformen, die sich der Grundlagenforschung, aber auch der Verknüpfung von Medizin und Technik, der Robotik und der künstlichen Intelligenz widmen. Sowohl bei der Entwicklung als auch bei der klinischen Implementierung innovativer Techniken sind Chirurgen gefordert, nicht nur als Operateure, Wissenschaftler oder Ärzte, sondern in der integrativen Rolle eines „physician scientist“ oder „surgeon scientist“. Hierzu braucht es von der DGCH gemeinsam mit den Fachgesellschaften etablierte Strukturen.

Chirurgische Forschung ist jedoch in erster Linie klinische Forschung – hierbei sind zwei Aspekte besonders wichtig:

1. Chirurgische Forschung ist keineswegs auf Universitätskliniken beschränkt, ganz im Gegenteil.
2. Die Chirurgie muss in der klinischen Forschung umdenken, sie muss größer denken.

Auch wenn nicht alle chirurgischen Fragestellungen einer prospektiven Untersuchung zugänglich sind, so sollte zukünftig der Fokus der klinischen Forschung noch deutlich stärker als bisher auf kontrollierte, nach Möglichkeit randomisierte Studien gelegt werden. Wenngleich wissenschaftlich häufig weniger originell als die Grundlagenforschung, so sind diese klinischen Arbeiten doch von überragender Bedeutung für die Patientenversorgung.

Die DGCH hat hier mit der Gründung des Deutschen Studienzentrums und dem Chir-Net, dem größten Studiennetzwerk Deutschlands überhaupt, einen ersten, sehr wichtigen Schritt in die richtige Richtung gemacht. Allen Bereichen der chirurgischen Forschung ist gemein, dass es entsprechender struktureller und personeller Voraussetzungen bedarf. Hier liegt ein großes Problem, denn in wohl kaum einem Stellenplan findet sich für die chirurgische Forschung eine bedarfsgerechte Personalbemessung.

Aber genau diese ist für eine erfolgreiche Forschung eine Conditio sine qua non!

Nun ist die DGCH nicht in erster Linie eine Forschungsinstitution, sondern eine Vereinigung spezialisierter Fachgesellschaften mit eigenständigen wissenschaftlichen Profilen und Strukturen. Die DGCH kann aber, mehr als eine einzelne Fachgesellschaft es für sich alleine könnte, die essenzielle Bedeutung der chirurgischen Forschung herausstellen, gerade im sich wandelnden Kontext der Interdisziplinarität und im Vergleich mit den forschungsstärkeren konservativen Fächern. Das bereits erwähnte, initial mit öffentlichen Mitteln geförderte Deutsche Studienzentrum ist ein gutes Beispiel, wie wichtig die chirurgische Interessenvertretung für die Forschungsfinanzierung ist.

Eine solide Forschungsstruktur und -kultur sind dabei nicht nur für die fachliche und wissenschaftliche Weiterentwicklung der Chirurgie unabdingbar, sondern auch für die Attraktivität des Faches selbst. Und genau diese ist wichtig für die Nachwuchsgewinnung und -förderung.

## Nachwuchsförderung

Hier hat der Gründungsvater Theodor Billroth eine nahezu zeitlose Handlungsmaxime hinterlassen.

„Unsere Aufgabe ist es, die Gegenwart und soweit unser Blick reicht, die Zukunft unserer nächsten Generation nach unseren Kräften, nach unserem besten Wissen und Gewissen glücklich zu gestalten.“

Gute Lehre dient nachweislich der Nachwuchsakquise und ist daher eine Investition in die nächste chirurgische Generation. Es muss gelingen, junge Menschen für die Chirurgie zu faszinieren, sie für die Chirurgie zu gewinnen und sie im Laufe ihrer Ausbildung zu motivieren und zu fördern.

Die Deutsche Gesellschaft für Chirurgie hat für den Nachwuchs schon vor mehreren Jahren eine eigene Plattform, das sog. „Perspektiv-Forum“, etabliert. Diese ermöglicht dem Nachwuchs eine aktive Teilhabe an der Entwicklung der DGCH und auch eine Mitsprache bei Entscheidungen des Vorstandes und des Präsidiums. Aufgabe dieses Forums ist es zum einen, das Gebiet der Chirurgie bereits den Medizinstudierenden nahezubringen, und zum anderen, die Weiterbildungsassistenten auf ihrem Weg zum Facharzt, glücklicherweise immer häufiger zur Fachärztin, und zur weiteren Spezialisierung innerhalb des Fachgebietes zu begleiten.

In allen Umfragen zu Arbeitsbedingungen besitzen bei angehenden Chirurginnen und Chirurgen das Arbeitsklima und eine strukturierte operative Weiterbildung den höchsten Stellenwert. Hinzu kommt die Vereinbarkeit von Freizeit, Familie und Beruf. Und genau dort muss der chirurgische Nachwuchs abgeholt und seinen Vorstellungen, seinen Ideen und Idealen mit Vertrauen begegnet werden.

Mehr als 60 % der Studierenden sind weiblich, der Anteil an Mitarbeiterinnen in der Chirurgie erreicht aktuell jedoch nicht einmal 30 %. In den Leitungsebenen liegt er sogar nur im einstelligen Prozentbereich. Die Förderung des weiblichen Nachwuchses ist eine der vordringlichsten Aufgaben der Chirurgie und somit der DGCH. Es gilt, Arbeits- und Berufsbedingungen zu schaffen, die die immer noch bestehenden beruflichen Ungleichheiten der Geschlechter egalisieren. Die Chirurgie muss sich auf die Frauen einstellen und nicht umgekehrt. Auf diesem Weg ist die Chirurgie bereits ein gutes Stück vorangekommen. Der Anteil der Frauen wächst auf allen Stufen der Karriereleiter, einschließlich der Chefarztetage.

Das Ziel ist allerdings erst dann erreicht, wenn Familienplanung und Kinderbetreuung für niemanden mehr ein Hindernis für den beruflichen Werdegang sind, und es selbstverständlich ist, dass Frauen in der Chirurgie gleichberechtigt in führende Positionen aufsteigen.

## Ökonomisierung der Medizin

Der demografische Wandel sowie der medizinisch-technische Fortschritt haben in den letzten Jahren zu einem starken Kostenanstieg im Gesundheitssystem geführt. Dies erfordert auch in der medizinischen Versorgung zunehmend die Ausrichtung des Handelns an ökonomischen Aspekten. Wirtschaftlich sein bedeutet in erster Linie, begrenzt zur Verfügung stehende Ressourcen bestmöglich einzusetzen. Dies ist weder neu noch ungewöhnlich, noch ist es ethisch verwerflich. Im Gegenteil, eine Rationalisierung dient dem bestmöglichen Einsatz nur begrenzt zur Verfügung stehender Ressourcen. Ein wirtschaftlicher, vernunftbezogener Umgang mit begrenzten Ressourcen dient einer optimierten Verwendung dieser und entspricht somit zutiefst ethischen Ansprüchen.

Nicht verhandelbar hingegen ist das Primat des Medizinischen über das Wirtschaftliche. Medizin darf kein Geschäft werden! Einer „Kommerzialisierung“ der Medizin, einer Orientierung primär an Profit und finanziellem Nutzen muss die Chirurgische Gesellschaft strikt entgegenwirken.

Gerade die chirurgischen Disziplinen sind von der zunehmenden Kommerzialisierung und den Fehlanreizen eines an Prozeduren orientierten Vergütungssystems besonders betroffen. Eine Leistungssteigerung allein durch eine Erhöhung der Fallzahlen, nicht aber durch eine Verbesserung der Qualität wäre ein fataler Fehlschluss! Es ist eine der vordringlichsten Aufgaben der DGCH, auch mit Blick in die Zukunft und auf den Nachwuchs, für eine faire und gerechte finanzielle Abbildung der chirurgischen Leistungen einzutreten.

Die Preise von heute bestimmen bekanntlich die Strukturen von morgen!

Die dramatischen Vorhersagen für die ökonomische Entwicklung von Krankenhäusern werden zu einer Restrukturierung der Krankenhauslandschaft in Deutschland führen. Hier sei beispielsweise der „Krankenhausplan NRW“ genannt, der bereits konkrete Vorstellungen für die Schaffung spezialisierter Leistungsbereiche mit besonderen Qualitätsanforderungen enthält. Bei allen genannten Umstrukturierungen, die über die wirtschaftlichen Folgen hinaus zudem auch mit weitreichenden Auswirkungen für Lehre, Aus- und Weiterbildung einhergehen, muss die DGCH fest eingebunden sein und ihre Stimme erheben – als Advokat ihres Faches, ihrer Mitglieder und ihrer Patienten!

Die Zukunft der Chirurgie ist verknüpft mit der Zukunft unserer Gesellschaft, so kann auch die die Zukunft der DGCH nicht losgelöst von der gesamtpolitischen, der sozialen, der ökonomischen und insbesondere auch der ökologischen Entwicklung gesehen werden. Nur wer auf der Höhe seiner Zeit ist, wird angemessene Antworten auf deren Fragen finden.

Getragen von den 10 Säulen der Fachgesellschaften und querverwoben durch 9 Arbeitsgemeinschaften und 3 Sektionen ist die DGCH die Heimat aller Chirurgen Deutschlands. Für die Erfüllung der jetzigen und zukünftigen Aufgaben der DGCH bedarf es des Schulterschlusses aller unter ihrem Dach vereinten Kräfte, einschließlich deren Bereitschaft, Partikularinteressen für den übergeordneten Erfolg auch einmal zurückzustellen. In der aristotelischen Gewissheit, dass „das Ganze mehr ist als die Summe seiner Teile“, werden wir nur als geeinte Gemeinschaft die Zukunft unseres Faches erfolgreich gestalten können. Die Deutsche Gesellschaft für Chirurgie wird dabei die Stimme der gesamten Chirurgie sein, vernehmbar, verlässlich und immer dem Wohle des Patienten verpflichtet.

